# Factors Influencing Staff Support for Sensory Device Use for People in Long-Term Care Settings

**DOI:** 10.1177/07334648251377485

**Published:** 2025-09-19

**Authors:** Najwan El-Saifi, Carly J. Meyer, Naomi Rose, Kasia Bail, Colette Browning, Dayna R. Cenin, Melanie Ferguson, Chyrisse Heine, Lisa Keay, Sheela Kumaran, John Newall, Emma Scanlan, Hamid R. Sohrabi, Melinda Toomey, Johanna Westbrook, Piers Dawes

**Affiliations:** 1 1974 The University of Queensland Centre for Hearing Research (CHEAR), The University of Queensland, Brisbane, Australia; 2 232133 Bolton Clarke Research Institute, Brisbane, QLD, Australia; 3 Centre for Healthy Ageing, Health Futures Institute, 5673 Murdoch University, Perth, WA, Australia; 4 Centre of Ageing Research and Translation, 2234 University of Canberra, Canberra, ACT, Australia; 5 1458 Federation University Australia, Mt Helen, VIC, Australia; 6 95974 Brightwater Research Centre, Perth, WA, Australia; 7 1649 Curtin University, Perth, WA, Australia; 8 1458 Federation University Australia, Ballarat and Gippsland, VIC, Australia; 9 7800 University of New South Wales, Sydney, NSW, Australia; 10 Department of Linguistics, Faculty of Medicine, Health and Human Sciences, 7788 Macquarie University, Sydney, NSW, Australia; 11 104167 Hearing Australia, Sydney, Australia; 12 Australian Institute of Health Innovation, 7788 Macquarie University, Sydney, Australia

**Keywords:** hearing/vision impairment, dementia, long-term care, aged care, hearing aids

## Abstract

Residents in long-term care settings do not receive adequate support for hearing and vision devices. This study aimed to identify factors influencing support for residents’ daily use of hearing and vision devices. Semi-structured interviews with 23 staff were analyzed deductively to categorize data within the Capability, Opportunity, and Motivation model of Behavior (COM-B), and then inductively to identify themes and subthemes within each domain guided by the Theoretical Domains Framework. Capability factors included a lack of skills in managing and maintaining sensory devices, interpersonal communication skills, and forgetting about device use. Opportunity factors included device management not being included on care task lists and inaccessible sensory devices. Staff reported a strong sense of duty and positive feelings about providing device support, reflecting high motivation.

**What this paper adds**

• This study identifies behavioral factors contributing to sensory device support in long-term care at the individual, interpersonal, and organizational levels.
• It provides recommendations to improve hearing and vision care in long-term care settings.

**Applications of study findings**

• Interventions should include training to equip staff with the knowledge and skills for effective support for hearing aids and glasses.
• Sensory device support tasks should be integrated into staff daily care routines.
• There is a need to establish best practice guidelines for sensory device support and management in long-term care settings.

## Introduction

Hearing and vision impairments are highly prevalent among older people living in long-term care settings, with approximately 90% experiencing hearing impairment and over 50% experiencing vision impairment (Bowen et al., 2016; Cohen-Mansfield & Taylor, 2004). Hearing and vision impairments negatively impact physical, psychosocial, mental, and cognitive well-being (Mick et al., 2014; Tseng et al., 2018). Vision impairment, for instance, limits independence, daily functioning, and mobility (Gallagher & Jackson, 2012), as well as increasing the incidence of disorientation, confusion, and falls (Rees et al., 2009; Stelmack, 2001). Hearing impairment similarly impacts quality of life, causing communication difficulties, loneliness, social isolation, and frustration (Bennett et al., 2022; Heffernan et al., 2022). When hearing and vision impairments coexist “dual sensory impairment,” difficulties are even more pronounced (Yamada et al., 2014). With the high prevalence of dementia in long-term care affecting up to 75% of residents (Gordon et al., 2014), hearing and vision impairments can further exacerbate responsive behaviors, leading to greater social isolation, increased medication use, use of physical restraints, and higher care costs (Heine & Browning, 2014; Leroi, 2020). Furthermore, hearing and vision impairments impact paid carers and family members resulting in depression, exhaustion, relationship stress, and increased care burden among care partners (Lawrence & Murray, 2009). Hearing and vision interventions (e.g., hearing aids and glasses) are effective in improving communication and reducing depressive symptoms and loneliness among older adults (Dawes et al., 2018; Punch & Horstmanshof, 2019) and improving outcomes for people living with dementia (Leroi et al., 2020). Addressing hearing and vision impairment is therefore important for optimizing quality of life for older people.

Despite the high prevalence of hearing and vision impairments among people in long-term care settings, these impairments are often undertreated (Andrusjak et al., 2020; Leroi et al., 2020). Many residents do not receive adequate support, including with hearing aids (e.g., insertion, battery changes) and glasses, environmental adjustments (e.g., noise reduction, improved lighting), and communication strategies (e.g., speaking face to face) (Andrusjak et al., 2020; Cross et al., 2022). As older people often experience reduced dexterity, many residents may struggle to manage their own hearing aids, with most residents relying on staff for hearing aid support (Punch & Horstmanshof, 2019). This dependence is even more pronounced for residents with dementia (Bott et al., 2022). A recent review highlighted several barriers to the identification and management of sensory loss in long-term care settings (Andrusjak et al., 2020). These included challenges with use and maintenance of assistive aids (e.g., hearing aids and glasses), limited access to specialists such as audiologists and optometrists, “unfriendly” sensory environments (e.g., high levels of background noise), reduced cognitive capabilities among residents, and a lack of staff knowledge about prevalence, impact, and support for hearing and vision impairment (Andrusjak et al., 2020). While training to improve staff knowledge about hearing aid maintenance may support hearing aid use, some studies reported that hearing aid use among people with hearing loss in long-term care remains low (Cohen-Mansfield & Taylor, 2004; Cross et al., 2024b). Although some interventions have focused on staff training, they show mixed results and have addressed only a narrow range of factors (Cohen-Mansfield & Taylor, 2004; Cross et al., 2024b). Broader, more comprehensive interventions are lacking, representing a significant missed opportunity to improve residents’ quality of life.

To support the hearing and vision needs of long-term care residents, it is therefore essential to understand the factors that influence staff in providing day-to-day support for sensory device use. These factors include those related to staff, residents, the local environment, and the broader care ecosystem. We used a health behavioral model to describe these factors: the Capability, Opportunity, and Motivation model of Behavior (COM-B) (Michie et al., 2011). According to the COM-B model, for a behavior to occur, individuals must possess physical and psychological capability (e.g., skills and stamina), have physical and social opportunities (e.g., time and social norms), and be motivated both reflectively and automatically (e.g., beliefs and emotions) (Michie et al., 2011). When a desired behavior does not take place, it may be that barriers in one or more of these COM-related areas need to be addressed (Michie et al., 2011). The COM-B model underpins the Behaviour Change Wheel Framework, which guides the design of evidence-based, theory-driven interventions targeting behavior change. By identifying and addressing COM-B components, interventions become more effective in promoting desired behaviors (Michie et al., 2011). This has been previously applied to shape health care professional behaviors across different contexts (Barker et al., 2018; Bennett et al., 2023).

The aim of this study was to identify and describe the factors influencing long-term care staff support for residents’ daily use of hearing and vision devices for residents according to the COM-B model. By systematically mapping these factors using a behavior change framework, this research will provide the foundational evidence needed to develop targeted interventions that enhance staff support for sensory device use in long-term care settings.

## Methods

### Study Design and Setting

This qualitative study utilized semi-structured interviews with staff working in one long-term care facilities in Perth, Australia. The aim was to identify factors that influence how staff support day-to-day device use which involved the following aspects:

• Each morning, check glasses are on and clean.

• Each morning, check that hearing aids are on, inserted correctly, on correct ears, and working.

• Each evening, check hearing aids and personal listening devices are removed, wiped clean, and stored safely and batteries are conserved.

• Each week, check frames are well aligned and in good order.

The interview topic guide is provided in supplementary material. This study and related findings are reported in line with the consolidated criteria for reporting qualitative research (COREQ) (Tong et al., 2007). Ethical clearance for this research was granted by the Human Research Ethics Office of The University of Queensland (Ethics ID number: 2022/HE001477).

### Sample

Long-term care staff were approached directly by senior managers at two sites of the facility. Staff were eligible to participate if they were currently working in long-term care in Australia and provided daily care support to residents, were able to provide informed consent, and self-reported adequate conversational English. Maximum variation purposive sampling was applied to ensure diversity across roles (e.g., nurses, healthcare assistants, and managers), seniority levels, and ethnicity.

### Data Collection and Analysis

Interviews were conducted face to face by a trained research assistant (N.R.) at the long-term care setting where participants worked between May 2023 and August 2023 and lasted between 30 and 60 min. Participants completed a demographic questionnaire at the beginning of the interview, and with their permission, the interviews were audio-recorded.

Recordings were transcribed verbatim, de-identified, and verified for accuracy against the original digital recordings. Data were analyzed using a hybrid analytic approach, combining deductive (COM-B-guided coding) with inductive (data-driven thematic) analysis. Three members of the research team (N.E.S., C.M., and N.R.) independently conducted a deductive analysis to map potential factors influencing day-to-day device use support to the COM-B model: Capability (physical and psychological), Opportunity (physical and social), and Motivation (automatic and reflective). Subsequently, two researchers (N.E.S. and C.M.) conducted an inductive thematic analysis, following Braun and Clarke’s method (Braun & Clarke, 2006). This included familiarization with the data, generation of initial codes, development of themes and subthemes, and iterative refinement. These themes were then aligned, where possible, to the 14 domains of the Theoretical Domains Framework (TDF) (e.g., knowledge, social influences, environmental context, and resources) (Atkins & Michie, 2015) to more easily inform implementation strategies. Where TDF domains were too broad to fully capture specific findings (e.g., “Social influences” did not sufficiently capture nuances such as “residents’ reactions to staff support”), new themes were generated to preserve the richness of the data. Microsoft Excel was used to organize and interpret the data within the COM-B and emergent themes. Independent coding was cross-checked, and any discrepancies were discussed until resolution was reached between the two researchers (N.E.S. and C.M.). The characteristics of the participants were summarized using descriptive statistics.

## Findings

Twenty-three participants were recruited for the study. Recruitment continued until no additional attributional themes emerged and data saturation was reached. Most participants were female (91%) with an average age of 42 years, primarily spoke a language other than English (74%), and had over 5 years of experience working in long-term care (61%) (Table 1). Key roles included care workers (43%), nurses (26%), and allied health professionals (22%).

**Table 1.** Sample demographics (*n* = 23)

Demographics	*n* (%)
Age in years—mean (SD)	42 (13)
Female	21 (91)
Born overseas	19 (82)
Languages other than English	17 (74)
Work title	
Care worker	10 (43)
Nurse	6 (26)
Allied health professional	5 (22)
Manager	2 (9)
Qualifications	
Secondary school education	2 (9)
Diploma, certificate or equivalent	7 (30)
Bachelor’s degree or equivalent	10 (43)
Postgraduate qualification (master’s or doctoral degree)	3 (13)
Other qualification	1 (4)
Years in profession	
Less than 2	5 (22)
2–5	4 (17)
5–10	5 (22)
More than 10	9 (39)

The analysis revealed 21 core themes within the COM-B. Themes and subthemes are presented in Table 2. Exemplar quotations from participants are used to support the themes. Participants are indicated by number (i.e., P_1_).

**Table 2.** Summary of themes and subthemes

COM-B domain themes	Subthemes	Quotes
*Psychological capability*		
Knowledge and skills	• Knowledge of residents’ hearing and vision needs • Knowledge of impact of dementia on residents	• P_3_: *“So it comes with time to also know the care plans and know the routines so that when you go to do your checks, you know that it's done, versus sometimes you don’t know everyone. It’s hard to learn 60 residents at once.”* • P_20_: “*Well the dementia client will not be able to tell you if their hearing aid is dirty, need cleaning or it’s not working. All you can see most of the time is behaviour, especially where I work. If I get a client, I know if he depends on his hearing aid and it’s not actually insert very well, definitely there’s going to be...So I think they really need our support and education of... And when you put it on, because over the years I’ve noticed people put it on nicely but they don’t know whether it’s on. Whether it’s actually on.”*
	• Device management skills • (Lack) Interpersonal skills • Skills learnt with experience	• P_6_: “*No, I use my shirt or whatever. I don’t come across it very often.”* • P_11_: “*We can’t do anything. Just report to the nurses. We can’t really do much if they don’t want it.”* • P_1_: “And it’s not written down anywhere, it’s just kind of learned on the job, that that’s how you’d do it.”
Memory, attention, and decision processes	• Noticing when devices aren’t worn or need to be cared for • Forgetting to support daily device use	• P_15_: “*And sometimes I go to the care staff and I said, for example, ‘[Name] is not wearing her hearing aids’. And they say, “Oh, okay.” So they go and have a look*.*”* • P_2_: “*Yes. Sometimes when it’s rushed, we forgot to put the hearing aid as well. And then we realize, oh, okay, we forgot to give him or her hearing aid, and we just go.”*
Behavioral regulation	• Offering flexible hearing and vision support to meet residents’ needs	• P_1_: “*Yeah. So if they don’t want to have them at that particular time, you go back later, maybe after 10 minutes, and check if they would prefer not to have them, or*
*Physical opportunity*		
Time	• Staff are too busy to support device use	• P_22_: *“Overall I think the pressure that staff are put under to complete their roles all contributes to them not always being compliant with this.”*
Barriers	• Device-related factors • Resident-related factors	• P_15_: “*So they go and have a look. They’re either missing or they are no charge or there’s different staffing that don’t know if she wears hearing aids.”* • P_20_: *“…Put the nasal prongs and-Yeah. So every time, his ears used to be a little bit red and those were... Very often, well you can’t do anything about it, but we used to put cream just to keep it dry and moist and all that sort of thing.”*
Prompts and cues	• Observing clues in the resident’s room prompts staff to support device management • Residents request their devices and assist staff in putting them on • Device management needs to be on the task list to get done	• P_1_: “*And also, there’s also like poster things in everyone’s room, that wears glasses and hearing aids, just to let them know that they need glasses and hearing aids.”* • P_11_: “*React. I think they’re also used to it. Even if we forget, they usually tell us, ‘I need to put my hearing aids on’, so yeah.”* • P3 “*We don’t have a set task order of how to do things. It’s more just you read the care plan, you see what needs to be done for their morning routine, and you just kind of get that done the best that you can.”*
Material resources	• Hearing aid guidance • Training in device management • Availability and accuracy of sensory information in care plans • Policies and procedures governing sensory care and device management	• P_7_: “*Because some of them got an instruction. Yes. Some of them. And then they just like, you can’t have, can’t see. But some of them is easy to know. Okay. Easy to adjust. Yeah. You just put it and fit properly.”* • P_11_: “*I think they need more training with the hearing aids rather than this, because I think we usually get trained to communicate with these, just how to manage, I think, the hearing aids better because some of them get lost sometimes.”* • P_22_: *“And then hopefully they read their care plan, which would have the information in it for them to be informed around the individual.”* • P3: “*I’d have to look into what the clinical protocols are, because I can’t think of a very specific protocol off the top of my head.”*
Accessibility	• Accessibility of care plan • Availability and accessibility of maintenance kits and cleaning products • Staff ability to find devices in residents’ rooms • Having a designated place for staff to store hearing and vision devices • Labeling of devices is important	• P_3_: “I think we’re looking to update that system because we have a lot of agencies who come in, and they don’t know the care plans, and then they don’t have access to the care plan.” • P_22_: “*So I think, around this, as a weekly check, I would ensure that hearing aids have sufficient supply of batteries, if they’re used, or that a check of the charging device was made. So those pieces of equipment that support the aid, that supports the client, also need to be reviewed regularly.”* • P_6_: *“But then some people I think either misplace them or stash them somewhere and don’t quite remember where they’ve stashed them for safekeeping, and they can be a little bit trickier to find.”* • P_6_: “*For some residents, usually the ones that are a bit more cognitively able, they’re typically in the bedside table or the top drawer, so that’s fine.”* • P_7_: “*I wish the name is there. Because sometimes they mix up this one, taking this one. Even the staff think. Oh, that’s her glasses. No, that’s not the one. They’re just guessing.”*
*Social opportunity*		
Cultural norms	• Cultural, language, and accent do (not) impact day-to-day device management • Cultural and religious beliefs guide quality care	• P_17_: “*So we had someone today that, he keep on saying, ‘What did you say?’ Maybe that’s also sometime a language barrier, but we need to explain.”* • P_23_: “A *lot of them come from that background where elderly people are their main focus in their families. We don’t prioritize our age care the way that some other people do. Even Italians and Greeks, moms and mothers and fathers and grand grandparents, they’re very highly regarded.*”
Organizational culture	• Senior management support day-to-day device use	• P_17_: “*Very supportive. We had a recent management actually, taking action straight away.”*
Interpersonal influences	• Residents need assistance to wear their hearing and vision devices • Residents don’t know what they need and then don’t know what help to ask for • Residents misplace their own or others’ hearing and vision devices • Family members make decisions that are not in the best interests of the residents • Staff are eager to support residents with device use, but they prioritize residents’ preferences and autonomy • Residents’ inability to see or hear prompt staff to support device use and find lost devices • (No) peer pressure	• P_20_: “*And this was a person that can actually independent walk around. I thought that she can’t... No matter she can eat by herself or go around, but her hearing aid, she depends on it only with carer’s assistance.”* • P_7_: “*They don’t have, and then they don’t ask they need, as you know, I don’t know. I just don’t want to go too much.”* • P_7_: “*And someone, some of the resident, because our facilities there are dementia, they go in someone’s drawer, they taking things, the same thing you know, I told you. The glasses, she take it and she hide. everyone be looking everywhere not find after a week.”* • P_15_: *“[Name], the family didn’t buy. I’m pretty sure they stopped buying the hearing aids because she will take them out.”* • P_1_: “*Some of them don’t like hearing aids, though they need hearing aids. So you kind of go with what they want, at the end of the day. Just document it.”* • P_7_: “*This person was upset, aggressive, and yelling because he can’t hear. That’s why he think we can’t hear him. He’s like, I can’t, Do you listening to me. I can’t hear what you say. He’s very upset. When the hearings come, he put it, he’s hearing, he’s so good.”* • P_12_: “*Yeah, the nurses, they do. But in particular, since there wasn’t any issue in related to this, I think they didn’t also highlight or make emphasis on it, I think.”*
Residents’ reactions	• Residents are (NOT) happy to receive support for using their hearing and vision devices • Residents’ reactions to device use support and their ability to autonomously put on their hearing aids and glasses depend on the level of their cognition, dementia, and mood • Residents take off the hearing aids after they put them on or after the support and misplace them	• P_3_: “*Sometimes they want to do it on their own, and you know if they do it on their own, they’re not going to do it properly. So you kind of have to judge how much assistance you’re going to give. And that kind of comes from knowing the person. If they’re fighting you because you are putting their glasses on, then you let them put it on, and then you just have to do your quick check without interfering too much knowing that it's still in properly.”* • P_20_: “*Well, it depends on the mood. Sometimes it’s good to leave. Sometimes it’s just good to leave and then come back with a new approach. Sometimes the approach is another thing.”* • P_16_: “*It’s very hard with the dementia people because dementia people sometimes they don’t like to wear the glasses. They throw it anywhere. They put it, they don’t know where’s the glasses. They lost their glasses. They lost their hearing aids. They get annoyed with the sound that comes from hearing aids. They don’t like to put it.”*
Social support	• Extent of collaboration between staff • Peer role modeling to support daily device use • Staff check devices are on (working and clean) and prompt device support if it hasn’t been done • Sharing information about residents’ device needs and device management from colleagues • Staff do not fulfill their responsibilities regarding daily support for device use • Seeking specialist support from colleagues for day-to-day device management • Staff communicate with families to help meet residents’ device management needs • Families prompt device use and management	• P_9_: “*Well, whatever the residents needs are, we’re happy to provide for them, because we’re here for the residents. We’re here to support each other work as a team, but if there’s any things to be followed up on, we have to hand it over to the afternoon shift for the following day.”* • P_12_: “*And then, yeah, that’s what we do. What my friends also doing, that’s what I learned from them at that time.”* • P_12_: “*If the charge is exhausted again, something has wrong, even the carers didn’t check on him, like he’s hearing aids, we just go and check and then just plugged in and put it in charge.”* • P_17_: “*Because often our nurse, RN will just let them know that say, this particular client had a hearing aid, and they had a specific instruction how to put on and why the reason is, the resident’s had an infection, so they have specifically on care plan. So we had to make sure they read the care plan, especially if there is a specific information for that client. Yes, we do have to making sure that they have been trained how to put it on. And a staff member will carry the weight, to help to train them, how to apply the hearing aid. Eye glasses okay, but the hearing aid, sometimes got different kind of type of hearing aids.”* • P_23_: “*The evenings, to be honest, I think it gets missed. Okay. I think they’re not taking them out. They’re certainly not cleaning them. They’re certainly not wiping them clean, and they’re definitely not opening up that battery door.”* • P_16_: “*That’s our job because RN have to report forward or RN have to refer them according to their needs.”* • P_9_: “*One resident here, the frame of the glass got broken from this part, so you can’t really put them ... We’ve got a first aid repair for the time being, because it will not work. It would end up scratching the resident’s face, so we just ended up putting it aside, informed family, and it took a while for the family to get another replacement.”* • P_17_: “*But you need to remind yourself that this is it, she has got that in the morning, that you need to apply it, because the family member will not going to be happy with you, and they will be complain. So it all be onto us. So making sure that you don’t forget, you forget, so you say sorry about it.”*
*Automatic motivation*		
Emotions	• Experiences negative emotions when not able to fulfill support needs • Experiences positive emotions when about to fulfill support needs • Feeling frustrated when other staff don’t do what they should to support device use • Feeling comfortable supporting daily device use	• *P2*: *“It frustrates me, when something doesn’t work. To be honest, I get really frustrated.”* • P_9_: *“Well, whatever the residents needs are, we’re happy to provide for them, because we’re here for the residents.”* • P_10_: *“You feel uncomfortable when you find the resident is wearing hearing aids you did not do, but someone did. He did not check the battery or he did not check if it is broken, then the battery’s in the hearing aids, but it’s not changed. It’s a week, it’s supposed to be changed, but they did not change it. And then you see someone is uncomfortable, it makes you... The day is not okay, this, this, this, until you figure out what is happening to today, what is going on? And then when you ask, ‘Yeah, the battery is changed and everything’. ‘When we go to check, you find the problem was the battery is not on and the resident cannot hear’. Then I was like, why the resident cannot hear? So when I change the battery, put a new one and put it on, ‘Can you hear me?’ ‘Yes’. Ah, this was the problem. They did not change the battery. But from morning until afternoon, I was not happy, he doesn’t even talk to someone.”* • P_19_: “*No, I’m always comfortable because you learn by yourself how to do it*.*”*
Impulses: Habit and routine	• Supporting device use is (not) part of the regular routine	• P_12_: *“Well, in the evening when we go to bed we make sure their hearing aids are plugged in, make sure it’s charging and then we also take off the glasses. Sometimes if there’s time we just make sure the glasses are clean and put it back in the case and yeah, that’s it.”*
*Reflective motivation*		
Goals and intentions	• Supporting device use “has to be done”	• P_1_: “*Yes. It has to be done. It’s not a matter of, ‘Oh, I’ll put it after breakfast’, it just has to be done.”*
Optimism	• Varying levels of optimism regarding the likelihood of residents wearing hearing and vision devices	• P_23_: “*I think it’s helping. I’m getting less complaints from family. It’s not the clients that complain, it’s the families. So I’m getting a few quite a few less complaints. So I think that’s working.”*
Beliefs about consequences	• Supporting device use helps residents to hear and see • Supporting device use improves residents’ communication, health, and well-being • Supporting device use improves residents’ safety	• P_14_: “*It impacts everything. I know if they can’t see, then he goes, “I can’t see. I can’t watch TV, I can’t read, I can’t.”* • P _20_: “*And you can hear the sound if it’s on and everything, so that the client can hear you when you speak. So that helps with the communication between you and the client. Because if a client is using an hearing aid and you don’t put it on or you apply it and it’s not working, there’s no point because you get behaviors, because he’s not hearing whatever you trying to explain to him.”* • P_12_: *“And when you can see properly and when you see the other residents just couldn’t see anything and just fell off, it’s not fair for them to have broken their wrists or their ankles. At least you should be kind enough to help the older people to at least live their life like us.”*
Beliefs about capabilities	• Care staff are (NOT) confident in managing hearing and vision devices	• P_23_: “*And I think that could also come with confidence as well. You’ve got a dozen or so, or half a dozen that are quite confident that might say that. But I would say the biggest majority would say, oh, okay then. And just leave it. And then when family comes and say, he hasn’t got his hearing aids, he, but he didn’t want to put them in. And so family are going, well, I don’t care what he wanted. It’s your job to make sure he is got them in so he can hear*.”
Professional role and identity	• Supporting device use is considered part of their role as team leader • Supporting device use and knowledge of who needs this support is considered part of their professional role • Device maintenance is considered part of the role of care staff	• P_10_: “*But I always, because as a team leader, I have to make sure even if they’re having lunch or breakfast in the morning, I have to check sure someone have a denture in, glasses on, hearing aids in.”* • P_12_: *“If I see the particular residents who has dementia walking without the glasses or the hearing aid, it’s my duty to make sure that she’s got everything that she need.”* • P_21_: *“But we need to be very careful if they’re wearing hearing aids, we need to take off before shower, before sleep. And just where we are placing it. It’s a very expensive thing, definitely. It’s our responsibility. We can’t say that it's client’s responsibility, it’s also our responsibility. We need to be very careful.”*

### Psychological Capability

#### Knowledge and Skills

Staff knowledge of residents’ hearing and vision needs varied. Some were unaware of whether residents owned hearing aids or glasses. Conversely, other staff were well-informed about which residents use glasses or hearing aids, and the health conditions (e.g., dementia) that affect their use. These knowledgeable staff were also aware of factors related to hearing aid use for individual residents, including those that refused to wear them, those that frequently misplaced their hearing aids, or those in need for assistance, particularly among those with dementia. They highlighted how dementia impacts residents’ ability to manage their hearing and vision devices, leading to challenges such as forgetting to wear them, residents not being able to check if devices were working properly or being able to use them.

P_23_: “I don't think it's coming from a lack of care. I think it's more, oh, I didn't know he wore hearing aids, or I didn't know they wore hearing aids.”

P_20_: “Well the dementia client will not be able to tell you if their hearing aid is dirty, need cleaning or it's not working. All you can see most of the time is behavior.”

Staff attributed this inconsistency in knowledge to staffing arrangements. They explained that agency staff, who may only work once or twice a week, have limited familiarity with residents, while those who work more consistently know the residents better. Participants also added that new staff have less knowledge than those who have been working for longer periods and have developed a deeper understanding of the residents’ needs over time.

P_15_: “Most of the carers, it's a lot of changing of different carers. So, it's not like a routine that carer knows the client.”

Staff expressed a lack of knowledge and skills pertaining to device management. Knowledge of hearing aids was challenging, due to the variety of types and the insufficient guidance provided, as many devices lacked clear user manuals or instructions. While some staff reported that they were comfortable with basic tasks such as checking if hearing aids were in the correct ears and whether they were functioning, they lacked knowledge of more advanced tasks like changing batteries, properly using the devices, and inserting them correctly. Cleaning of glasses and hearing aids was also problematic, with staff unsure of appropriate cleaning methods, often resorting to using tissues or personal clothing. Staff were unsure how to check the frames and screws of glasses. Consequently, problems with devices were only addressed once they were broken or not working.

P_22_: “I think there's a lack of knowledge around how to change batteries, how to use the devices, how to insert them correctly. It's quite often, not until something like a pair of glasses actually break or something happens to them, that it's escalated and, as a result, actioned.”

Staff also noted limited understanding of the importance and value of hearing aids, further hindering effective device management.

Despite these challenges, some staff reported developing their skills through experience.

P_10_: “They never teach us. So, we learn on job.”

Many participants highlighted their interpersonal skills and how these were important in supporting device use. Staff reported that they showed empathy and adapted communication strategies based on individual resident needs, particularly for those with dementia. They employed strategies including demonstrating actions to explain how devices worked and showing the devices to residents beforehand to ensure understanding. For residents who did not want to put on their glasses or hearing aids, staff reported trying gentle persuasion. Staff reported that a personalized approach—such as suggesting that a person would need their glasses to complete a crossword puzzle—was effective in encouraging residents to use their sensory devices.

Some staff highlighted using gestures, visual cues, and mimicking actions or pointing to help residents with comprehension difficulties. They demonstrated how to use hearing aids or glasses, and when hearing aids were broken, they relied on written communication or speaking loudly.

P_9_: “Because sometimes we just have to make visual cues for them to understand what we're trying to do for them.”

Some staff reported a lack of communication skills, often failing to relay or document residents’ sensory care needs, such as lost glasses during handovers.

P_7_: “I think it need a little bit more communication because one person got glasses, meet that person, need his glasses. And then if they don't have it, where is the glasses? Where it go?.. They need assist. That's why when the glasses have to be in the iCare (the electronic care record system), whatever, in the handover.”

#### Memory, Attention, and Decision Processes

Staff reported observing residents’ hearing and vision difficulties through facial expressions or by noticing dirty, crooked, or broken glasses.

Most staff reported frequently forgetting daily tasks related to sensory support, including changing hearing aid batteries, putting on hearing aids, and conducting checks for functioning. Forgetting to carry out sensory support tasks was attributed by staff to feeling rushed or under time pressure.

P_2_: “Yes. Sometimes when it's rushed, we forgot to put the hearing aid as well. And then we realize, oh, we forgot to give him or her hearing aid, and we just go.”

#### Behavioral Regulation

Staff reported needing to manage other responsibilities while adjusting their work to accommodate extra time needed to support residents with dementia, ensuring flexible hearing and vision support.

P_16_: “Also, we re-approach them after a while, 10, 15 min, 20 min because they change quickly. And they might be next minute they say, ‘Oh, thank you so much for giving my glasses,’ and they're happy.”

### Physical Opportunity

#### Time

Staff believed that there is not enough time in their busy work schedules to support hearing and vision device management, or to check each resident’s care plan in relation to sensory needs. Lack of time is exacerbated by understaffing, changes in staff, and at the start of shifts.

P_22_: “Yes [to times when staff might forget to include device support in their routine] if they're under pressure, if they're feeling rushed, if they're running on lower than normal staffing levels, if there's been an event in the area that morning.”

#### Barriers

Participants discussed several barriers to supporting device use related to residents’ health conditions, including hair and skin conditions that made it difficult to wear hearing aids and glasses. Other barriers included poor fit of glasses and hearing aids, breakages, malfunctioning hearing aids due to wax buildup, hearing aid batteries being flat, and challenges with keeping hearing aids dry (e.g., during showering).

P_17_: “Because one of our clients, she had a excoriation skin problem on the ear...also, she had hearing aid and glasses at the same time.”

#### Prompts and Cues

Staff highlighted the absence of a clear task list to guide the daily management of hearing and vision devices, making it challenging to consistently meet residents’ needs. Some participants mentioned that including hearing support tasks (e.g., put on hearing aids) into the electronic task list system Clinical Manager was helpful and enabled more consistent support for hearing aids. Although staff colloquially referred to the system as “iCare,” Clinical Manager is the current electronic health record platform used by all clinical staff to record and access task lists, care plans, handover notes, and progress documentation.

Additionally, staff reported that some residents will remind staff about their hearing aids or request assistance with them. Staff also mentioned visual prompts within residents’ rooms, such as posters or small signs with notes about hearing aids:

P_2_: “Right now, they're putting in every room who have the hearing aids, the small notes, like please help me with my hearing aid, so that we know who have a hearing aid, who doesn't have.”

#### Material Resources

Staff reported that resident care plans are essential references for sensory needs, such as whether a person wears glasses or hearing aids, though some noted that hearing or vision information on care plans may not always be current. New staff reported that they learn from both the care plans and experienced colleagues, with registered nurses providing instructions about hearing aids and giving hands-on training.

P_22_: “And then hopefully they read their care plan, which would have the information in it for them to be informed around the individual.”

Although staff reported that they knew where to find information in the care plans and commented about the importance of regularly checking care plans, staff reported that they do not consistently take the time to read and use this information. Additionally, staff reported limited awareness of clinical protocols and procedures for managing hearing and vision impairment, as well as for assisting residents.

P_5_: “I'm not sure about that [policies and procedures] with the [Name aged care setting].”

Several participants reported a lack of formal training about supporting residents with hearing aids, stressing the need for such training. Some mentioned the availability of hearing aid manuals; while some aids come with clear, user-friendly instructions, others do not.

P_10_: “No, actually about the hearing aids and the glasses, we don't have a video. We don't have it. They never teach us. We learn on job.”

#### Accessibility

Staff reported challenges in discovering where each resident’s glasses and hearing aids were stored because storage locations differed. For residents with cognitive impairment or dementia, devices were typically stored in medication cupboards. Other residents usually keep their glasses or hearing aids in their rooms, often on bedside tables or in drawers. Some residents misplaced their devices. Staff changes meant that new staff did not know where a resident’s glasses or hearing aids were kept. Staff emphasized the need for a consistent storage place, particularly for residents with visual impairments, because people with visual impairments found it more difficult to find their devices.

P_6_: “Some people I think either misplace them or stash them somewhere and don't quite remember where they've stashed them for safekeeping.”

Another issue that affected accessibility of glasses and hearing aids was the lack of labeling. Most devices were not labeled with the residents’ names, so that staff did not know who they belonged to.

P_7_: “I wish the name is there. Because sometimes they mix up this one, taking this one. Even the staff think. Oh, that's her glasses. No, that's not the one. They're just guessing.”

Additionally, participants identified accessibility of care plans as a barrier to readily accessing information about residents’ sensory needs. Managers also discussed the challenges associated with changing from paper-based care plans to electronic systems. A shortage of iPads and laptops and technical problems limited access to electronic care plans. Agency staff do not have access to electronic care plans, hindering continuity of care. Staff reported that electronic care plans are not easy or quick to use, cannot be printed for reference, and do not provide concise summaries of care needs. These challenges mean that staff do not feel they have an easy way to be informed about residents’ needs.

P_22_: “They all have access to be able to do it, but it's not user-friendly. They have to go out of their way to access it and the system is not quick to give them the information.”

### Social Opportunity

#### Cultural Norms

Managers reported that effective care depends not only on the confidence and knowledge of staff but also on their cultural and language ability.

P_22_: “I think differences in levels of understanding of English, being culturally and linguistically diverse, can put people off reading if there's too much information there. So, we try to keep it simple.”

Some staff expressed a sense of religious duty, which guides them to perform their tasks.

P_19_: “God is watching us. So, I'm fear of God, I do not fear of human being.”

#### Organizational Culture

Staff described the work culture as positive, reporting that managers are supportive of the provision of sensory care, and informally educate staff about support for hearing and vision needs. Staff reported that managers were approachable and encouraged staff to seek support and assistance whenever they needed.

P_3_: “Say someone needed assistance in how to put hearing aids in or the type of stuff that they should be looking for to know if the hearing aid/glasses are working, the managers are always great at taking that opportunity to educate you.”

#### Interpersonal Influences

Participants recognized a need to assist residents with dementia in using their hearing aids and vision devices. Residents with dementia often forgot about their devices, lacked insight into their hearing or vision difficulties, and struggled with checking and using devices properly.

P_16_: “And then we always tell them, ‘You need your glasses if you want to read that book’. Some of them they understand, some of them they don't know what's going on.”

Some residents with cognitive impairment frequently misplaced glasses or hearing aids, took others’ glasses or hearing aids, or hid them. Consequently, families may refuse to provide hearing aids due to the cost of having to replace lost devices.

P_15_: “The family didn't buy. I'm pretty sure they stopped buying the hearing aids because she will take them out.”

Although nurses reported encouraging the use of hearing and vision devices, there was uncertainty about whether hearing and vision care policies were effectively monitored or enforced.

P_22_: “Are they [hearing and vision care policies and procedures] enforced? They should be.”

#### Residents’ Reactions

Residents with dementia require more time and patience to provide support. Their reactions to assistance with hearing aids or glasses were reported to vary depending on cognitive level, dementia stage, and mood.

P_16_: “It's very hard with the dementia people because dementia people sometimes they don't like to wear the glasses. … They get annoyed with the sound that comes from hearing aids.”

To address these challenges, participants emphasized the need for adaptability, for example, returning later that day and trying a different strategy. Staff noted that insisting too strongly on using glasses or hearing aids could lead to verbal or physical aggression.

P_3_: “Unfortunately, sometimes it's physical aggression. Sometimes it's verbal. So, you just have to be aware that if someone's going to get aggressive because of it.”

#### Social Support

Most staff highlighted a strong sense of team spirit and support, with carers working collaboratively with nurses and therapist assistants to ensure that hearing aids and glasses are functional and clean. Morning handovers facilitated communication. Staff reported seeking specialist support from colleagues when dealing with issues such as broken glasses or malfunctioning hearing aids and consulting occupational therapy teams for repairs. Some staff reported that evening shift staff may not consistently perform device support tasks, such as turning off hearing aids overnight so that the batteries are not flat by the morning. Inconsistent overnight device support created additional challenges for day staff.

P_23_: “The evenings, to be honest, I think it gets missed. I think they're not taking them out. They're certainly not wiping them clean, and they're definitely not opening up that battery door.”

Participants added that families play a crucial role, advising staff on how to carry out daily checks or make repairs. Although repairs are usually quick, delays can occur if families are unavailable, or if the next of kin are slow to provide consent for repairs or replacement. Some families provide additional resources such as batteries or cleaning kits. Staff reported that families would raise concerns about sensory care, including lost glasses, or the cost of maintaining sensory aids.

P_23_: “The complaints that I'm getting from family again, is we're going through too many batteries, etcetera.”

### Automatic Motivation

#### Emotions

Staff expressed positive emotions such as happiness, satisfaction, and a sense of accomplishment in relation to supporting the hearing or vision needs of residents. However, they also expressed feelings of frustration, sadness, stress, and guilt when they felt that a resident’s sensory needs were not being met.

P_17_: “The residents are happy and you get the needs that they have met, then you be able to go home happy.”

#### Impulses: Habit and Routine

Most staff indicated that supporting device use is part of their daily routine. In the morning, staff routinely check hearing aids and glasses. Some staff clean the devices before helping residents put them on, while others clean them only if they notice they are dirty. At night, some staff would remove hearing aids, take out batteries, and turn off the devices or place them on chargers. Staff reported that there is no formal routine for checking glasses, but they would report loose or broken frames to the registered nurse in charge.

P_16_: “Just like we put in a routine. That person need a glasses, that person need a hearing aids. We have routine, we know how we work.”

### Reflective Motivation

#### Goals and Intentions

Some participants emphasized that assisting with day-to-day device use is part of their duty and that sensory care is an essential part of carer responsibilities.

P_11_: “This is also a responsibility, so we have to do it.”

#### Optimism

Staff were keen to support the use of hearing and vision devices, but they reported that this was often challenging. There were varying levels of optimism regarding the likelihood of residents using these devices regularly.

P_3_: “... we would love for people to wear their glasses, and we'd love for them to wear their hearing aids, but it's just sometimes not possible.”

#### Beliefs About Consequences

Participants believed that supporting hearing aid and glasses use was beneficial to residents, helping improve communication, enabling residents to participate in conversations and group activities, and fostering social engagement and happiness. For residents with dementia, staff reported that supporting device use helped relieve agitation among residents, improved communication with residents, and strengthened relationships between residents and staff.

P_15_: “Some of the clients that they get very agitated, they can't see, they can't actually read. So, it causes them to be upset.”

Staff also talked about how supporting device use improves residents’ safety, preventing falls and fractures, avoiding accidents, and maintaining independence.

P_1_: “They can hear us properly, they can see things properly, which can avoid a lot of falls. So, it benefits them a lot. And it saves us, from shouting in their ear.”

#### Beliefs About Capabilities

Staff confidence varied significantly in supporting hearing aid and glasses use, with many lacking confidence due to insufficient knowledge and training. Less confident carers would offer sensory support but readily accept resident refusal, lacking the confidence to appropriately influence resident choice.

P_23_: “You've got a dozen or so... that are quite confident... But I would say the biggest majority would say, oh, okay then. And just leave it.”

This contributed to inconsistent device use and family concerns about unmet sensory needs.

#### Professional Role and Identity

Participants emphasized that supporting device use and knowing which residents need assistance is a key part of their professional role.

P_10_: “And I'm not here to do because of someone. I'm doing it because it's part of my job and I love... You know how they could say it, they say duty of care.”

## Discussion

Our findings suggest that long-term care staff perceive a need to support hearing aid and glasses use and are motivated to provide that support but experience numerous challenges. Staff reported a lack of confidence and need for formal training in how to support hearing aid and glasses use. The workplace was reported to be positive and supportive of provision of sensory care: however, there were challenges with a lack of awareness of institutional hearing and vision care policy and practice, difficulties accessing information about sensory needs in residents’ care plans, and time pressures in delivering care. Staff turnover and time constraints were identified as factors contributing to a lack of familiarity with residents’ individual needs, which negatively impacted the consistency of sensory support. Sensory needs were sometimes neglected because they were not well integrated into care routines or communicated between staff. Cognitive impairments, such as dementia, create challenges due to residents’ fluctuating abilities, emphasizing the need for tailored support.

Our findings are consistent with previous studies identifying barriers such as lack of knowledge and low confidence among long-term care staff in supporting use of assistive hearing/vision devices, along with lack of formal training in hearing or vision care (Andrusjak et al., 2020; Cross et al., 2024a; Dawes et al., 2021; Punch & Horstmanshof, 2019). Additional barriers include suboptimal management of sensory aids, unsuitable environment, lack of communication among the team, and challenges associated with addressing sensory needs of residents with cognitive impairment. Importantly, it has been acknowledged that the barriers to providing sensory support to residents are complex and that collaborative multifaceted approaches are required. A supportive work environment and team collaboration are essential for effective device use. Furthermore, partnerships with families by involving them in care planning and educating them about the benefits of consistent device support and the impact of unmet sensory needs encourage their active role.

These findings align with the limited intervention literature identified in our introduction, highlighting the need for multifaceted interventions. While prior intervention studies have primarily focused on staff training for hearing aid maintenance with mixed outcomes (Cohen-Mansfield & Taylor, 2004; Cross et al., 2024b), our systematic exploration using the COM-B framework offers insights into why such narrowly targeted interventions may have had limited success. Specifically, our findings highlight that training alone (targeting *capability*) is insufficient when barriers related to *opportunity* (e.g., inaccessible devices and time constraints) and *motivation* (e.g., lack of confidence) remain unaddressed. This helps explain the variable results of prior interventions and reinforces the need for a comprehensive, multifaceted approach (Meyer et al., 2025).

Based on our findings, we propose the following seven recommendations to improve support for hearing and vision device use in long-term care settings: First, provide staff training and skill development focused on practical aspects of device use, such as insertion, cleaning, and troubleshooting. This addresses psychological capability gaps in knowledge, limited training access, and enhances confidence. Second, embed hearing and vision care tasks into daily routines and electronic documentation systems (e.g., checklists, care plans) to ensure consistency across shifts. This supports behavioral regulation, routine building, and reduces omissions due to time pressure or forgetfulness. Third, introduce visual prompts and reminders in resident environments and care plans to support memory, habit formation, and continuity of care during busy periods.

Fourth, ensure consistent storage and labeling of devices, and improve access to maintenance materials. This enhances physical opportunity by reducing device loss, confusion, and inconsistent placement of aids. Fifth, strengthen team communication and handovers and foster collaboration with families. This supports social opportunity by improving interpersonal coordination and reinforcing staff motivation through positive feedback and shared responsibility. Sixth, clarify and monitor institutional policies and ensure timely access to up-to-date care plan information to support consistent practice. Seventh, establish stronger partnerships and more consistent collaboration with the internal occupational therapy department to ensure specialized assessments and ongoing support, particularly for complex cases. This builds on existing team-based practices and addresses the need for accessible clinical expertise. Collectively, these recommendations address the multi-level factors identified in this study and provide a foundation for improving sensory support as part of routine aged care.

### Limitations and Strengths

A key strength of this study is the use of the COM-B model and TDF, which provided a systematic, theory-informed approach to identifying factors that influence how staff support device use in long-term care. This enabled an in-depth exploration of the behavioral drivers underlying hearing aid and glasses use, thereby informing the design of targeted interventions.

As this study was conducted in a single long-term care facility, these findings may not be representative of all long-term care settings. However, a strength of the study was the in-depth insights obtained directly from aged care staff. The diversity across participants’ roles and employment levels offered a range of perspectives on daily challenges, skills, and knowledge gaps. This has contributed to a more comprehensive and inclusive understanding of the barriers and enablers to supporting hearing and vision devices in aged care. Although deductive analysis may limit findings to COM-B components and TDF domains, combining deductive analysis with inductive thematic analysis allowed us to uncover broader themes. This combined approach enhanced analytic rigor while capturing nuanced insights beyond the theoretical framework.

## Conclusion

Staff in long-term care settings face multiple challenges with supporting daily use of hearing aids and glasses among residents despite being motivated to do so. These challenges span individual knowledge and skills, environmental and institutional constraints, and communication processes. Addressing these factors requires a multi-level approach that includes staff training, integration of sensory care into routine workflows, improved documentation systems, and clarification of responsibilities across clinical roles.

Multi-level interventions are therefore necessary to target factors at the individual, interpersonal, and organizational levels to improve quality of care for residents. Findings highlight the need for policy frameworks and best-practice guidelines that include sensory support as a core component of high-quality aged care. Future research should evaluate the implementation of targeted, behaviorally informed strategies and explore their impact on staff practice and resident outcomes across diverse care contexts.

## Supplemental Material


**Supplemental material -** **Factors Influencing Staff Support for Sensory Device Use for People in Long-Term Care Settings**
Supplemental material for Factors Influencing Staff Support for Sensory Device Use for People in Long-Term Care Settings by Najwan El-Saifi, Carly Meyer, Naomi Rose, Kasia Bail, Colette Browning, Dayna R. Cenin, Melanie Ferguson, Chyrisse Heine, Lisa Keay, Sheela Kumaran, John Newall, Emma Scanlan, Hamid R. Sohrabi, Melinda Toomey, Johanna Westbrook, and Piers Dawes in Journal of Applied Gerontology

Supplemental material - Factors Influencing Staff Support for Sensory Device Use for People in Long-Term Care Settings
Supplemental material for Factors Influencing Staff Support for Sensory Device Use for People in Long-Term Care Settings by Najwan El-Saifi, Carly Meyer, Naomi Rose, Kasia Bail, Colette Browning, Dayna R. Cenin, Melanie Ferguson, Chyrisse Heine, Lisa Keay, Sheela Kumaran, John Newall, Emma Scanlan, Hamid R. Sohrabi, Melinda Toomey, Johanna Westbrook, and Piers Dawes in Journal of Applied Gerontology
