# Supplementary material for: Factors Influencing Staff Support for Sensory Device Use for People in Long-Term Care Settings
Source: J Appl Gerontol. 2025 Sep 19;45(8):1530–45. doi: 10.1177/07334648251377485 (PMC13323916; doi:10.1177/07334648251377485)
Supplement: Supplemental material - Factors Influencing Staff Support for Sensory Device Use for People in Long-Term Care Settings [file sj-pdf-1-jag-10.1177_07334648251377485.pdf]

COREQ (COnsolidated criteria for REporting Qualitative research) Checklist

A checklist of items that should be included in reports of qualitative research

Topic	Item No.	Guide Questions/ Description	Author Responses
**Domain 1: Research team and reflexivity**			
Personal Characteristics			
Interviewer/facilitator	1	Which author/s conducted the interview or focus group?	Ms Naomi Rose
Credentials	2	What were the researcher's credentials? E.g. PhD, MD	Bachelor of Science (Speech Pathology) (Honours)
Occupation	3	What was their occupation at the time of the study?	Speech Pathologist (SP)
Gender	4	Was the researcher male or female?	Female
Experience and training	5	What experience or training did the researcher have?	Previous research experience facilitating a communication intervention with people with dementia and collecting their baseline and follow up data. Worked as a SP with various populations of people with cognitive impairment, including in residential aged care facilities.
Relationship with participants			
Relationship established	6	Was a relationship established prior to study commencement?	No
Participant knowledge of the interviewer	7	What did the participants know about the researcher? e.g. personal goals, reasons for doing the research	Nothing
Interviewer characteristics	8	What characteristics were reported about the inter-viewer/facilitator? e.g. Bias, assumptions, reasons and interests in the research topic	The interviewer was a health clinician trained as a qualitative researcher. No specific biases, assumptions, or prior relationships with participants were reported. The interviewer's interest in the research topic was informed by professional experience in health care and a focus on improving sensory care in long-term care

[OFFICIAL]

			settings
**Domain 2: Study design**			
Theoretical framework			
Methodological orientation and Theory	9	What methodological orientation was stated to underpin the study? e.g. grounded theory, discourse analysis, ethnography, phenomenology, content analysis	Fully detailed in the Data Collection and Analysis section.
Sampling	10	How were participants selected? e.g. purposive, convenience, consecutive, snowball	Participants were selected using maximum variation purposive sampling.
Method of approach	11	How were participants approached? e.g. face-to-face, telephone, mail, email	Participants were approached directly by senior managers at two sites of the facility
Sample size	12	How many participants were in the study?	Twenty-three participants
Non-participation	13	How many people refused to participate or dropped out? Reasons?	There were no refusals or dropouts
Setting			
Setting of data collection	14	Where was the data collected? e.g. home, clinic, workplace	Data were collected at the long-term care setting where participants worked.
Presence of non-participants	15	Was anyone else present besides the participants and researchers?	No. Only the participant and researcher were present during interviews.
Description of sample	16	What are the important characteristics of the sample? e.g. demographic data, date	Sample characteristics are described under the findings section and summarized in Table 1
Data collection			
Interview guide	17	Were questions, prompts, guides provided by the authors? Was it pilot tested?	Yes, a topic guide was provided and is available in the supplementary material. The guide was pilot tested prior to data collection.
Repeat interviews	18	Were repeat interviews carried out? If yes, how many?	No repeated interviews were carried out.
Audio/visual recording	19	Did the research use audio or visual recording to collect the data?	Yes, interviews were audio recorded with participant consent.
Field notes	20	Were field notes made during and/or after the interview or focus group?	Fields notes were taken during the interviews as needed
Duration	21	What was the duration of the interviews or focus	Interviews lasted between 30-60 minutes

		group?	
Data saturation	22	Was data saturation discussed?	Recruitment continued until no additional attributional themes emerged and data saturation was reached
Transcripts returned	23	Were transcripts returned to participants for comment and/or corrections?	Transcripts were not returned to participants for comment or correction but were reviewed for accuracy against the original digital recordings
**Domain 3: analysis and findings**			
Data analysis			
Number of data coders	24	How many data coders coded the data?	Details were provided in Data collection and analysis section
Description of the coding tree	25	Did authors provide a description of the coding tree?	No formal coding tree was developed. The analysis combined deductive coding using the COM-B model and Theoretical Domains Framework with inductive thematic analysis following Braun and Clarke's approach. Themes and subthemes were identified and refined iteratively but were not organized into a hierarchical coding tree
Derivation of themes	26	Were themes identified in advance or derived from the data?	Themes were both deductively mapped using the COM-B model and TDF and inductively derived through thematic analysis following Braun and Clarke's approach. Fully detailed in the Data Collection and Analysis section.
Software	27	What software, if applicable, was used to manage the data?	No qualitative data software was used. Data were organized using Excel.
Participant checking	28	Did participants provide feedback on the findings?	No, participants did not provide feedback on the findings
Reporting			
Quotations presented	29	Were participant quotations presented to illustrate the themes/findings? Was each quotation identified? e.g. participant number	Yes. Participant quotations were presented to illustrate the findings, and each quotation was identified by participant number
Data and findings consistent	30	Was there consistency	Yes. Themes were grounded in the

		between the data presented and the findings?	data, and representative quotations were provided to support each finding.
Clarity of major themes	31	Were major themes clearly presented in the findings?	Yes, major themes were presented in the findings and supported with quotations.
Clarity of minor themes	32	Is there a description of diverse cases or minor themes?	Minor subthemes were included in table 3

Developed from: Tong A, Sainsbury P, Craig J. Consolidated criteria for reporting qualitative research (COREQ): a 32-item checklist for interviews and focus groups. *International Journal for Quality in Health Care*. 2007. Volume 19, Number 6: pp. 349 – 357

Additional Guidelines for Completing the COREQ Checklist for *Journal of Applied Gerontology*:

- This checklist will be published online as supplementary material and we require it to be in the form of a publishable table. Please make sure that material does not bleed outside of cells, etc.
- This checklist is designed to direct readers to relevant material in the manuscript. Where applicable, please direct readers to various sections of the manuscript, such as a Methods section, Conceptual Framework, table or figure. Pages may shift during the publication process so please avoid directing readers to specific page numbers.
- This checklist also is designed to supplement information that may not be reported in the text and/or provide additional details related to information that is reported in the text.

Once you have completed this checklist, please save a copy and upload an anonymized version of it as part of your *Journal of Applied Gerontology* submission. DO NOT include this checklist as part of the main manuscript document. It must be uploaded as a separate supplemental file. If the paper is accepted, a non-anonymized version should be provided with the final submission of the main manuscript.
