# Supplementary material for: Factors Influencing Staff Support for Sensory Device Use for People in Long-Term Care Settings
Source: J Appl Gerontol. 2025 Sep 19;45(8):1530–45. doi: 10.1177/07334648251377485 (PMC13323916; doi:10.1177/07334648251377485)
Supplement: Supplemental material - Factors Influencing Staff Support for Sensory Device Use for People in Long-Term Care Settings [file sj-pdf-2-jag-10.1177_07334648251377485.pdf]

Thank you for meeting with me today as part of the SENSEcog Care interviews.

We are implementing a hearing and vision support programme with staff and residents of [Site 1] and [Site 2] later this year. These interviews are part of the planning process to make sure we understand your challenges and deliver something practical and useful according to staff feedback.

There are no right or wrong answers to any of the questions, so please just say what you really think. You can skip over questions if you like, or answer in as much or as little detail as you like. We will remove your name and other personal information from the interview transcript to protect your identity.

Please let me know if you would like to take a break or stop the interview at any point.

We will be recording the interview so that we have an accurate record of what was said today. Are you happy to start the recording now?

We are trying to understand what staff already do, and what helps you do things well, plus we'd like to know what you don't do, and why. This will give us a good idea of what makes it easier for you to provide hearing and vision support, and what makes it harder.

Behaviour #1 is about assisting residents with their day-to-day management of hearing and vision devices. By that we mean:

- **Each morning, check residents glasses are on and clean**
- **Each morning, check residents hearing aids are on, inserted correctly and on correct ears, and working**
- **Each evening, check hearing aids and personal listening devices are removed, wiped clean, and stored safely and batteries are conserved (on charge / battery door open)**
- **Each week, check frames are well aligned and in good order (e.g., screws tight, nose pads intact, no corrosion of metal frames or fragmentation of plastic)**

Take a look at this list. To what extent do you do these things?

Great. Now just take another look at the list and let me know if there is anything else here you currently do?

How often? Particular time of day?

Do you think a person with dementia needs the same support with these tasks as a person without dementia?

What would you do differently when you are supporting a person with dementia to do these things?

	**Possible prompts**
**Psychological capability**<br><br>*Firstly, I'm interested in exploring how your knowledge and skills influence day-to-day device management ….*	• Do you know which residents in your facility have hearing or vision loss and use glasses and hearing aids? If you are not familiar with a resident, how do you find out this information?<br>• Talk me through how you would assist a resident put on a hearing aid if they needed help. [skills]<br>• How would you tell if a person's hearing device or glasses needed repair?<br>• Are there ever times when you forget to carry out these daily checks? Tell me more.
**Physical opportunity**<br><br>*Next I'm interested in how your physical environment influences day-to-day device management …*	• If someone is new to a caring role, how are they trained to provide this day to day support? *Is it something that is written down or part of usual procedures, or something that is learned on-the job?*<br>• Talk me though how assistance with device use for each resident fits into your normal (morning / evening / daily / nightly) routine? How are your morning/afternoon routines decided? How do you manage changes in routine.<br>• How might the physical set-up of residents' rooms impact day-to-day device management? *How easy is it to find glasses and hearing aids in a resident's room?*
**Social opportunity**<br><br>*Now let's turn our attention to the social environment in your workplace, referring to interpersonal influences and social and cultural norms …*	• How supportive is your direct line manager of you providing this assistance to residents each day? Tell me more.<br>• How supportive are your colleagues of you providing this assistance to residents each day?<br>• How do residents react when you carry out these daily checks? How do you manage these reactions?<br>• Tell us about any cultural considerations or cultural beliefs that might impact day-to-day device management.
**Reflective motivation**<br><br>*We're now going to focus on your attitudes and beliefs about assisting with day-to-day device management …*	• How does it benefit residents when you provide this assistance?<br>• Are there any downsides to assisting with device use?<br>• How do you balance assisting with day-to-day device use alongside your other responsibilities?
**Automatic motivation**<br>*Lastly, we're interested in how assisting with day-to-day device use makes you feel ….*	• How does providing this assistance SUCCESSFULLY make you feel? (What word would you use to describe the feeling)? If a resident won't allow you to assist them, how does that feel?<br>• Can you tell me about situations where you felt uncomfortable about assisting with device use?
	• Are there any other factors that we haven't covered?

Nearly finished!

Last question - Do you want us to send you a summary of the research results at a later date?

Thanks so much for your time today. Feel free to get back in touch with me any time if you have any questions or concerns about what we discussed today
